# Deformation law of overlying rock strata during the initial mining period of a coal mining face: mechanical analysis

**DOI:** 10.1038/s41598-025-28696-x

**Published:** 2025-12-29

**Authors:** Li Chuantian, Liu Zeyu, Liu Liting, He Yongliang, Fu Yuping, Sun Xiaoyuan

**Affiliations:** 1 School of Engineering for Safety and Emergency Management, Taiyuan University of Science and Technology, 030024 Taiyuan, Shanxi People’s Republic of China; 2 Intelligent Monitoring and Control of Coal Mine Dust Key Laboratory of Shanxi Province, Taiyuan University of Science and Technology, 030024 Taiyuan, Shanxi People’s Republic of China; 3 School of Applied Sciences, Taiyuan University of Science and Technology, 030024 Taiyuan, Shanxi People’s Republic of China

**Keywords:** Rock strata, Initial mining period, Mechanical analysis, Failure behaviour, Pressure relief, Energy science and technology, Engineering, Solid Earth sciences

## Abstract

The deformation and failure behaviour of overlying rock layers during the initial stage of coal seam mining directly affects the stability control of surrounding rock and the prevention of dynamic disasters in the mine. To address the unclear mechanism of overlying rock structural instability and insufficient quantitative characterization of the evolution process under high-gas and low-permeability coal seam mining conditions, mechanical models were established—based on the assumption of elastic foundation, beam deformation, and key layer theory—for the deflection curve and bending moment equations of key layer deformation during the initial mining period under two boundary conditions of broken and unbroken coal walls in the mining face. The relationships among displacement, bending moment, mining advance influence distance, stiffness, coal rock mechanical properties, and working face advancement are quantified. The calculation of the mechanical performance parameters reveals that the vertical displacement of the key layer above the solid coal in front of the coal wall alternately increases and decreases towards the deep part of the coal rock mass, with the peak value gradually decreasing and approaching zero. The influence distance of horizontal advance mining on the working face is positively correlated with the bending stiffness of the key layer. Under the boundary of the fractured zone, the peak value is smaller than that of the nonfractured zone because of the greater range of coal wall pressure relief in the fractured zone. The vertical displacement of the key layer above the goaf section increases as it moves farther from the coal wall and shows a polynomial variation with increasing distance from the coal wall. This study reveals the mechanical mechanism by which the deformation of key layers during the initial mining period influences the evolution of overlying rock fractures, providing a theoretical basis for achieving coal and gas co-mining.

## Introduction

As a prerequisite and main energy source for China’s economic prosperity and sustainable development, the safe and efficient mining of coal is the core issue in the field of overlying shale control^1,2^. As shallow resources gradually deplete, deep mining faces complex mechanical environments such as high ground stress and strong mining disturbances^3,4^. The noncoordinated deformation of overlying rock layers during the initial mining stage has become the main cause of major accidents, such as roof collapse and impact ground pressure^5,6^, posing serious challenges to coal mine safety and efficient mining and highlighting the urgency of studying the instability mechanism of overlying rock during this stage.

Scholars worldwide have conducted extensive research on the movement laws of overlying rock layers. Huo Shusen^7^ used a similar physical model to study the movement law of overlying rock layers under the influence of mining activities and divided the movement of overlying rock layers into different stages to provide a basis for rock stability in mining faces. Chen Long^8^ analysed the key layers and determined the damage characteristics of the overlying strata and the relationship between surface movement and deformation. Effective measures in the mining area can reduce surface deformation and settlement. Lin Hai^9^ studied the control mechanism of overlying strata using the continuous filling mining method and derived an equation for roof deflection and a criterion for roof instability. Wang Yuliang^10^ studied the failure mechanism and displacement law of overlying strata and constructed fluid structure coupling and overlying strata movement characteristics. Fan Kaifang^11^ proposed a dynamic device for identifying the separation of mining rock layers to determine the movement and evolution laws of overlying strata, and the results were used to investigate the settlement of overlying strata. Through numerical simulation, Zhang Defei^12^ studied the characteristics of overlying strata damage and ground subsidence caused by excavation of working faces and analysed the characteristics of overlying strata cracks, the height of crack zones, and the evolution of stress fields. Jiang Jinquan^13^ used UDEC numerical simulation to study the stress evolution of the working face and section, the movement characteristics of the overlying strata, and the fault slip law when the working face advances from the lower wall to the upper wall. Ren Zhaopeng^14^ used a new discrete element method to study the overlying rock layers and loose layers, obtaining the synergistic failure characteristics of mining damage under dual-medium conduction and analysing the conduction law of high-intensity mining damage in the western mining area under a dual medium. Zhang Jicheng^15^ used mechanical models and numerical simulations to analyse the movement patterns and pressure distribution characteristics of the overlying rock strata on a fully mechanized mining face. These results have important reference significance for the mining pressure law and disaster prevention under similar conditions. Li Zhu^16^ developed a new method based on the law of energy evolution to determine the height of the collapse zone and the damaged area of the roadway roof. The damage criterion was derived by calculating the strain energy of the basic volume. Yang Zhongping^17^ reproduced the complete process of rock fracture and slope instability under the conditions of descending and ascending mining in gently inclined coal seams through approximate model experiments.

Scholars worldwide mainly use numerical simulations and similar physical simulations to study the deformation law of overlying rock layers^18,19^ and rarely apply theoretical analysis. This article is based on the assumption of elastic foundation, beam deformation, and key layer theory and establishes mechanical models for the deflection curve and bending moment equations of key layer deformation during the initial mining period under two boundary conditions: broken and uncrushed coal walls at the mining face. Both broken and uncrushed mechanical models are represented by piecewise functions^20^. The relationships among displacement, bending moment, mining advance influence distance, stiffness, coal rock mechanical properties, and working face advancement are quantified. The results provide reference and technical support for further exploration of the transfer and evolution characteristics of strain energy during failure deformation to ensure safe mining, disaster monitoring, and prevention in fully mechanized mining faces with thick overlying rock layers.

## Mechanical model of deformation of key layer beams during the initial mining period

### Assumption of the structural mechanics model during the initial mining period

**Fig. 1:**
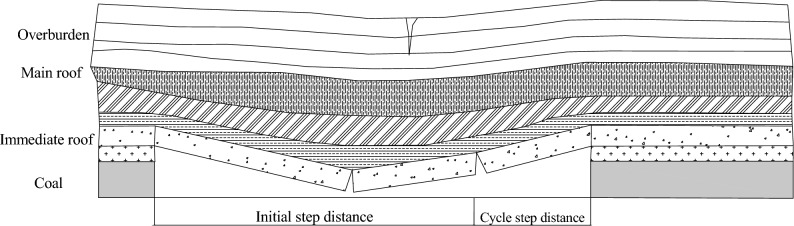
Schematic diagram of the overburden structure characteristics during the initial mining period.

On the basis of key layer control theory and the deformation characteristics of the overlying strata during the initial mining period^21^, a model study was conducted on the deformation of the overlying strata during the specific stage of the initial mining period of the mining face, as shown in Fig. 1.

#### Assumption of a rock beam structure

There are two main assumptions for the initial fracture study of the old roof in the mining area: one is to consider the roof as a rock beam structure, and the other is to consider the roof as a slab structure, which requires selecting the corresponding structural form for modelling according to different research objectives^22^. The rock beam structure is suitable for studying the fracture law of the roof along the working face direction in the middle, whereas the slab structure is suitable for studying the fracture law of the roof under the surrounding fixed support conditions of the entire working face. Previous studies have shown that the effect of overlying rock migration on the evolution of mining-induced fractures is concentrated mainly in the middle of the working face (middle of the goaf). Therefore, in the model study of the deformation of the overlying strata in coal mining faces, the key layer is assumed to be a rock beam structure.

During the initial mining stage of the working face, the cumulative footage is less than the length of the working face, and the fracture pattern of the main roof (key layer) presents a vertical “*X*” fracture, with the middle part penetrating along the inclination of the working face and occupying the advantageous position. The deformation of the middle part of the overlying rock strata along the direction of the working face has a significant controlling effect on the evolution of mining-induced fractures. During the initial mining period, the key strata of the overlying strata undergo bending deformation and subsidence before the initial pressure, which accounts for approximately 2/3 of the entire initial mining period. The deformation of key layers during the initial mining period is beam bending deformation.

#### Elastic foundation assumption

The ability of the working face coal as a boundary support point to restrict its downwards movement during the subsidence process of the key strata in the goaf is weak, and the range of its subsidence disturbance will penetrate deeply into the coal rock layer^23^. The mechanical model boundary of the key layer beam structure of the overlying rock during the initial mining period conforms to an elastic foundation.

Compared with the main roof (key layer), the adjacent surrounding rock has lower stiffness; in particular, the tensile strength of the coal seam is weaker, and it has a certain degree of elasticity. It can be approximately considered to comply with the assumption of an elastic foundation. The pressure of the overlying rock layer on any point of the foundation dominated by the coal seam is proportional to the settlement deformation of that point.

1 $$p = - k^{\prime}y$$

where $p$ is the pressure exerted by the overlying strata on the key layers, MPa. $k^{\prime}$ is the Winkler foundation coefficient, which is related to the mechanical properties of the supporting rock layers above and below the key layer. $y$ is the displacement of the key layer in the vertical direction, m.

#### Distribution law of the overburden load during the initial mining period

As the coal seam is extracted, the goaf area increases, and the overlying coal and rock layers bend and sink under the combined action of their own weight and the load of the overlying rock layers^24,25^. The rock layers produce mining-induced fractures, and the structural form and bearing capacity of the coal-bearing strata change. During the mining process, owing to the lower goaf, the separation or fracture of the upper overlying strata causes load transfer in the upper part of the key layer, and as the key layer bends and sinks, it is transferred to the coal body (surrounding rock support) in front of the working face and the collapsed rock blocks in the goaf.

The mechanical structures of the “simply supported beam” and “masonry beam” are used to control the movement law of the overlying strata before and after the key layer is broken. Under the action of mining, the load on the overlying strata in the mining area is usually not distributed uniformly. The load on the key strata of the overlying strata in the goaf is usually less than the original rock stress because of delamination and load transfer, whereas the load on the coal body in front of the working face is greater than the original rock stress because of the transmission of the overlying strata load.

### Theoretical basis for mechanical modelling

#### Mathematical modelling

To reveal the disturbance effect of key layer beam deformation on the surrounding rock during the initial mining period, it is necessary to obtain the deflection curve equation of beam deformation, determine the load concentration distribution law after beam deformation, and determine the maximum bending moment and corner position. This is accomplished on the basis of the relationships among deformation, internal forces, and load concentration of the bent beam^26^.

There is a straight beam AB, where *EI* is the flexural stiffness, and its endpoint position is determined by $x_{A} ,x_{B}$. When the deflection $\omega$, rotation angle $\theta$, bending moment *M*, shear force $F_{s}$, and load concentration *q* are all continuously differentiable, they satisfy the differential Eq. (1)

2 $$\frac{{d\left[ {EIW\left( x \right)} \right]}}{dx} = EI\theta (x),\frac{{d\left[ {EI\theta \left( x \right)} \right]}}{dx} = M(x),\frac{dM(x)}{{dx}} = F_{s} (x),\frac{{dF_{s} (x)}}{dx} = Fq(x)$$

If the functions $f_{0} (x),f_{1} (x)f_{2} (x) \ldots f_{n} (x)$ are continuous on [a, b] and differentiable on (a, b), then

3 $$\frac{{d^{n + 1} f_{0} }}{{dx^{n + 1} }} = \frac{{d^{n} f_{1} }}{{dx^{n} }} = \frac{{d^{n - 1} f_{2} }}{{dx^{n - 1} }} = \cdots = \frac{{df_{n} }}{dx} = f_{n + 1} (x)$$

The following is calculated:

4 $$f_{0} (b) - f_{0} (a) = \int_{a}^{b} {f_{1} (x)} dx = \mathop {\left[ {xf_{1} (x)} \right]}\nolimits_{a}^{b} - \int_{a}^{b} {f_{2} (x)} dx = \sum\limits_{i = 1}^{n} {\mathop {\left[ {\frac{{\left( { - 1} \right)^{i + 1} }}{i!}x^{i} f_{1} (x)} \right]}\nolimits_{a}^{b} } + \int_{a}^{b} {\frac{{( - 1)^{n} x^{n} f_{n + 1} (x)}}{n!}} dx$$

If

5 $$\sum\limits_{i = 1}^{n} {\mathop {\left[ {\frac{{\left( { - 1} \right)^{i + 1} }}{i!}x^{i} f_{1} (x)} \right]}\nolimits_{a}^{b} } - \int_{a}^{b} {\frac{{( - 1)^{n} x^{n} f_{n + 1} (x)}}{n!}} dx = 0$$

If $f_{n + 1} (x) = 0$, then there is

6 $$\sum\limits_{i = 1}^{n} {\mathop {\left[ {\frac{{\left( { - 1} \right)^{i + 1} }}{i!}x^{i} f_{1} (x)} \right]}\nolimits_{a}^{b} } = 0$$

The following relationship equation can be obtained from Eqs. (2) and (6):

7 $$\begin{gathered} \mathop {\left[ {EIw(x) - xM(x) + \frac{1}{2!}x^{2} M(x) - \frac{1}{3!}x^{3} F_{s} (x)} \right]}\nolimits_{{x_{A} }}^{{x_{B} }} - \int_{{x_{A} }}^{{x_{A} }} {\frac{1}{3!}x^{3} } q(x)dx = 0 \hfill \\ \hfill \\ \end{gathered}$$

8 $$\mathop {\left[ {EI\theta (x) - xM(x) + \frac{1}{2!}x^{2} F_{s} (x)} \right]}\nolimits_{{x_{A} }}^{{x_{B} }} - \int_{{x_{A} }}^{{x_{A} }} {\frac{1}{2!}x^{2} } q(x)dx = 0$$

9 $$\mathop {\left[ {M(x) - xF_{s} (x)} \right]}\nolimits_{{x_{A} }}^{{x_{B} }} + \int_{{x_{A} }}^{{x_{A} }} x q(x)dx = 0$$

10 $$\mathop {\left[ {F_{s} (x)} \right]}\nolimits_{{x_{A} }}^{{x_{B} }} - \int_{{x_{A} }}^{{x_{A} }} {q(x)} dx = 0$$

#### Relationships between deformation and external forces

AB is any two cross-sections of the beam, and its position is determined by $x_{A} ,x_{B}$. The external forces acting on the AB beam are analysed as follows: there are *r* concentrated force couples *m* (counterclockwise is positive), *s* concentrated forces *P* (upwards is positive), and *t* distributed loads *q* (upwards is positive). The location of the external force divides AB into *n* segments^27,28^.

$x_{i}$ represents the starting point coordinate $x_{i}^{ + }$ of the *i* beam, and the ending point coordinate of the *i-1* beam is $x_{i}^{ - }$.

11 $$x_{i}^{ + } = x_{i} + \varepsilon \left( {1 \le i \le n} \right),x_{i}^{ - } = x_{i} - \varepsilon \left( {2 \le i \le n + 1} \right)$$

where $\varepsilon$ is infinitely small.

From Eqs. (7)–(10),

12 $$\mathop {\left[ {EIw(x) - xEI\theta (x) + \frac{1}{2}x^{2} M(x) - \frac{1}{6}x^{3} F_{s} (x)} \right]}\nolimits_{{x_{i}^{ + } }}^{{x_{i + 1}^{ - } }} + \int_{{x_{i}^{ + } }}^{{x_{i + 1}^{ - } }} {\frac{1}{6}x^{3} } q(x)dx = 0$$

13 $$\mathop {\left[ {EI\theta (x) - xM(x) + \frac{1}{2}x^{2} F_{s} (x)} \right]}\nolimits_{{x_{i}^{ + } }}^{{x_{i + 1}^{ - } }} - \int_{{x_{i}^{ + } }}^{{x_{i + 1}^{ - } }} {\frac{1}{2}x^{2} } q(x)dx = 0$$

14 $$\mathop {\left[ {M(x) - xF_{s} (x)} \right]}\nolimits_{{x_{i}^{ + } }}^{{x_{i + 1}^{ - } }} + \int_{{x_{i}^{ + } }}^{{x_{i + 1}^{ - } }} {\frac{1}{2}x^{2} } q(x)dx = 0$$

15 $$\mathop {\left[ {F_{s} (x)} \right]}\nolimits_{{x_{i}^{ + } }}^{{x_{i + 1}^{ - } }} - \int_{{x_{i}^{ + } }}^{{x_{i + 1}^{ - } }} {q(x)} dx = 0$$

Owing to the continuity of $EIw$ and $EI\theta$ on the beam, then $EIw(x_{i}^{ - } ) = EIw(x_{i}^{ + } ),EI\theta (x_{i}^{ - } ) = EI\theta (x_{i}^{ + } )$.

At the point of concentrated force coupling, $m_{i} = M(x_{i}^{ - } ) - M(x_{i}^{ + } )$; otherwise, $M(x_{i}^{ - } ) = M(x_{i}^{ + } )$.

At the point of concentrated force coupling, $- P_{i} = F_{s} (x_{i}^{ - } ) - F_{s} (x_{i}^{ + } )$; otherwise, $F_{s} (x_{i}^{ - } ) = F_{s} (x_{i}^{ + } )$.

The starting point coordinate and the ending point coordinate of the distributed load $q_{j}$ are $x_{sj}$ and $x_{ej}$, respectively.

By superimposing Eqs. (12)–(15), the relationships between *w* and *θ* at sections A and B and the external force acting on beam AB can be obtained.

16 $$\mathop {\left[ {EIw(x) - xEI\theta (x)} \right]}\nolimits_{{x_{A} }}^{{x_{B} }} + \sum\nolimits_{j = 1}^{r} {\frac{1}{2}x_{j}^{2} m_{j} } + \sum\nolimits_{j = 1}^{s} {\frac{1}{6}x_{j}^{3} p_{j} } + \sum\nolimits_{j = 1}^{t} {\int_{{x_{sj} }}^{{x_{ej} }} {\frac{1}{6}x^{3} q(x)} } dx = 0$$

17 $$\mathop {\left[ {EI\theta (x)} \right]}\nolimits_{{x_{A} }}^{{x_{B} }} - \sum\nolimits_{j = 1}^{r} {x_{j} m_{j} } - \sum\nolimits_{j = 1}^{s} {\frac{1}{2}x_{j}^{2} p_{j} } - \sum\nolimits_{j = 1}^{t} {\int_{{x_{sj} }}^{{x_{ej} }} {\frac{1}{2}x^{2} q(x)} } dx = 0$$

18 $$\sum\nolimits_{j = 1}^{r} {m_{j} } + \sum\nolimits_{j = 1}^{s} {x_{j} p_{j} } - \sum\nolimits_{j = 1}^{t} {\int_{{x_{sj} }}^{{x_{ej} }} {xq(x)} } dx = 0$$

19 $$\sum\nolimits_{j = 1}^{s} {p_{j} } + \sum\nolimits_{j = 1}^{t} {\int_{{x_{sj} }}^{{x_{ej} }} {q(x)} } dx = 0$$

### Deformation mechanics model of key layer beams in the coal rock mass.

**Fig. 2:**
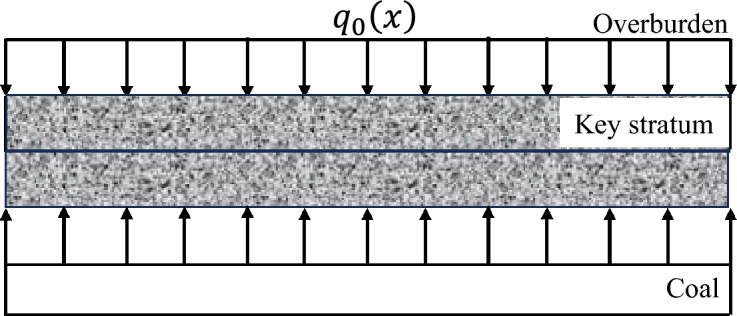
Load distribution during the initial mining period.

**Fig. 3:**
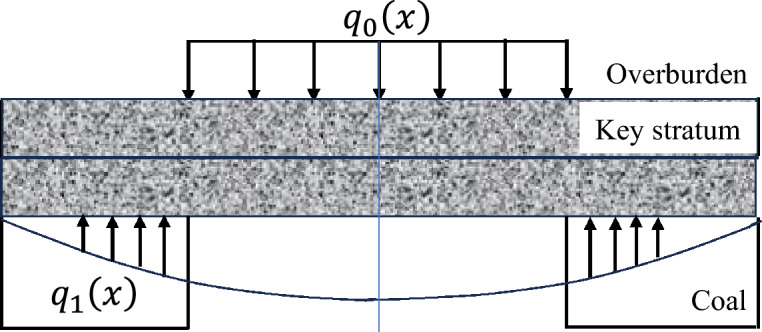
Diagram of the overburden load distribution in the noncrushing zone of the rib during the initial mining period.

**Fig. 4:**
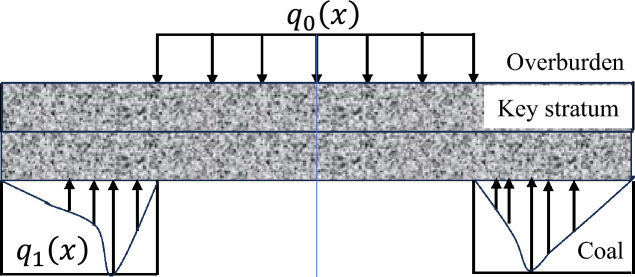
Diagram of the overburden load distribution in the crushing zone of the rib during the initial mining period.

The advance influence law of the elastic foundation boundary rock beam deformation of the key layer on the coal rock mass in front of the working face is determined within specific geological mining conditions and the deformation range of the key layer structure, including the advance influence range and load distribution law^29^. Considering the actual engineering situation, this mechanical model is modelled under two conditions: the absence of a fractured zone (unloading zone) and the presence of a fractured zone in the coal rock mass in front of the working face. The load distribution pattern at each stage of the initial mining period is shown in Figs. 2, 3, 4.

The deformation mechanics model of the elastic foundation boundary rock beam in the key layer without a fractured zone in front of the working face during the initial mining period is presented. The bending deformations of the overlying rock layer and the roof of the two longitudinal grooves in the coal mining operation, starting from the opening cut of the working face, are applicable to this mechanical model. The model takes the axis of the key layer rock beam as the x-axis, and the coordinate origin is selected at the original rock stress of the coal rock mass in front of the working face. The y-axis is perpendicular to the x-axis and passes through the coordinate origin. A is the horizontal distance from the stress point of the original rock in front to the coal wall of the working face, which is the influence area of key layer deformation on the advance of the coal body in front of the working face. B is the distance from the midpoint of the goaf towards the original rock stress point in front of the working face. Then, 2(b-a) is the span of the rock beam.

Within the range of 0 ≤ x ≤ a, the key layer is subjected to a downwards load from the overlying rock layer and an upwards load from the elastic foundation in opposite directions, with a magnitude equal to the difference between the two, denoted as $q_{1} (x)$. Within the range of a ≤ x ≤ b, the key layer is subjected to a uniformly distributed load $q_{0}$ from the overlying rock layer downwards, which is a constant.

Assuming that the deflection curve equation is $y = f(x)$, the following can be derived from Eq. (2) and the assumption of an elastic foundation beam:

20 $$\frac{{d^{4} y}}{{dx^{4} }} = - 4k^{4} y$$

where $k$ is a constant related to the mechanical properties of key layers and the elastic coefficient of the foundation.

The general solution of the high-order ordinary differential equation can be obtained from Eq. (19):

21 $$y(x) = (\cos kx,\sin ky)\left[ \begin{gathered} C_{1} {\kern 1pt} \;C_{2} \hfill \\ C_{3} \;C_{4} \hfill \\ \end{gathered} \right]\left( \begin{gathered} e^{kx} \hfill \\ e^{ - kx} \hfill \\ \end{gathered} \right)$$

The derivative is obtained from Eq. (21):

22 $$\frac{dy}{{dx}} = k(\cos kx,\sin ky)\left[ \begin{gathered} C_{1} + {\kern 1pt} C_{3} \;C_{4} - C_{2} \hfill \\ C_{3} - C_{1} \; - C_{2} - C_{4} \hfill \\ \end{gathered} \right]\left( \begin{gathered} e^{kx} \hfill \\ e^{ - kx} \hfill \\ \end{gathered} \right)$$

23 $$\frac{{d^{2} y}}{{dx^{2} }} = 2k^{2} (\cos kx,\sin ky)\left[ \begin{gathered} {\kern 1pt} C_{3} \; - C_{4} \hfill \\ - C_{1} \;C_{2} \hfill \\ \end{gathered} \right]\left( \begin{gathered} e^{kx} \hfill \\ e^{ - kx} \hfill \\ \end{gathered} \right)$$

$C_{1}$, $C_{2}$, $C_{3}$, and $C_{4}$ are determined on the basis of the following boundary conditions:

24 $${\text{When x}}\, = \,0{\text{and }}y(x)\, = \,0,{\text{then }}C_{1} {\kern 1pt} = - C_{2}$$

25 $${\mathrm{Whenx}}\, = \,0,\frac{dy}{{dx}} = 0,{\mathrm{and}}\frac{dy}{{dx}} = 0,{\text{ then }}C_{1} - C_{2} + C_{3} + C_{4} = 0$$

26 $${\mathrm{Whenx}}\, = \,0,\frac{{d^{2} y}}{{dx^{2} }} = 0,{\text{and }}\frac{dy}{{dx}} = 0,{\text{ then }}C_{3} = C_{4}$$

From Eqs. (24) to (26), the following conclusions can be drawn:$C_{2} = C_{3} = C_{4} = - C_{1}$.

When 0 ≤ *x* ≤ *a*

27 $$y(x) = (\cos kx,\sin ky)\left[ \begin{gathered} C_{1} {\kern 1pt} \; - C_{1} \hfill \\ - C_{1} \; - C_{1} \hfill \\ \end{gathered} \right]\left( \begin{gathered} e^{kx} \hfill \\ e^{ - kx} \hfill \\ \end{gathered} \right)$$

28 $$\frac{dy}{{dx}} = (\cos kx,\sin ky)\left[ \begin{gathered} 0\;C_{4} - C_{2} \hfill \\ - 2kC_{1} \;2kC_{1} \hfill \\ \end{gathered} \right]\left( \begin{gathered} e^{kx} \hfill \\ e^{ - kx} \hfill \\ \end{gathered} \right)$$

29 $$\frac{{d^{2} y}}{{dx^{2} }} = (\cos kx,\sin ky)\left[ \begin{gathered} {\kern 1pt} - 2k^{2} C_{1} \;2k^{2} C_{1} \hfill \\ - 2k^{2} C_{1} \; - 2k^{2} C_{1} \hfill \\ \end{gathered} \right]\left( \begin{gathered} e^{kx} \hfill \\ e^{ - kx} \hfill \\ \end{gathered} \right)$$

30 $$\frac{{d^{3} y}}{{dx^{3} }} = (\cos kx,\sin ky)\left[ \begin{gathered} {\kern 1pt} - 4k^{3} C_{1} \; - 4k^{3} C_{1} \hfill \\ \quad 0\;\quad \quad \quad 0 \hfill \\ \end{gathered} \right]\left( \begin{gathered} e^{kx} \hfill \\ e^{ - kx} \hfill \\ \end{gathered} \right)$$

31 $$\frac{{d^{4} y}}{{dx^{4} }} = (\cos kx,\sin ky)\left[ \begin{gathered} {\kern 1pt} - 4k^{4} C_{1} \;4k^{4} C_{1} \hfill \\ \quad 4k^{4} C_{1} \;4k^{4} C_{1} \hfill \\ \end{gathered} \right]\left( \begin{gathered} e^{kx} \hfill \\ e^{ - kx} \hfill \\ \end{gathered} \right)$$

As seen from Eq. (2).

32 $$EI\frac{{d^{3} y}}{{dx^{3} }} = \int {q_{1} (x)} dx$$

From Eq. (18), it can be concluded that

33 $$\int\limits_{0}^{a} {q_{1} (x)} dx + \int\limits_{a}^{b} {q_{0} (x)} dx = 0$$

Because $q_{0} (x)$ is a uniformly distributed load and a constant, $\int\limits_{a}^{b} {q_{0} (x)} dx$ is negative downwards and opposite in direction to $\int\limits_{0}^{a} {q_{1} (x)} dx$; therefore, Eqs. (2)–(33) give

34 $$\int\limits_{0}^{a} {q_{1} (x)} dx = \int\limits_{a}^{b} {q_{0} (x)} dx = q_{0} (b - a)$$

By solving Eqs. (34) and (32) simultaneously, if $0 \le x \le a$

$$EI\left[ {\frac{{d^{3} y}}{{dx^{3} }}} \right]_{0}^{a} = \left( {\cos kx,\sin kx} \right)\left[ \begin{gathered} - 4EIk^{3} C_{1} \quad - 4EIk^{3} C_{1} \hfill \\ \quad 0\quad \quad \quad \quad \quad 0 \hfill \\ \end{gathered} \right]\left( \begin{gathered} e^{ka} \hfill \\ e^{ - ka} \hfill \\ \end{gathered} \right) - (1,0)\left[ \begin{gathered} - 4EIk^{3} C_{1} \quad - 4EIk^{3} C_{1} \hfill \\ \quad 0\quad \quad \quad \quad \quad 0 \hfill \\ \end{gathered} \right]\left( \begin{gathered} 1 \hfill \\ 1 \hfill \\ \end{gathered} \right) = q_{0} (b - a)$$

The following is obtained:

35 $$C_{1} = \frac{{q_{0} (b - a)}}{{4EIk^{3} \left[ {2 - \cos ka\left( {e^{ka} + e^{ - ka} } \right)} \right]}}$$

Substituting Eq. (35) into Eq. (27) yields:

36 $$y\left( x \right) = \frac{{q_{0} (b - a)\left[ {\cos kx\left( {e^{kx} + e^{ - kx} } \right) - \sin kx\left( {e^{kx} + e^{ - kx} } \right)} \right]}}{{4EIk^{3} \left[ {2 - \cos ka\left( {e^{ka} + e^{ - ka} } \right)} \right]}}$$

If $$a \le x \le b$$

37 $$EI\frac{{d^{4} y}}{{dx^{4} }} = - q_{0}$$

38 $$EI\frac{{d^{3} y}}{{dx^{3} }} = - q_{0} x + D_{1}$$

39 $$EI\frac{{d^{2} y}}{{dx^{2} }} = - \frac{{q_{0} }}{2}x^{2} + D_{1} x + D_{2}$$

40 $$EI\frac{dy}{{dx}} = - \frac{{q_{0} }}{6}x^{3} + \frac{{D_{1} }}{2}x^{2} + D_{3}$$

41 $$EIy(x) = - \frac{{q_{0} }}{24}x^{4} + \frac{{D_{1} }}{6}x^{3} + \frac{{D_{2} }}{2}x^{2} + D_{3} x + D_{4}$$

If $$x = a$$.

42 $$EI\frac{{d^{3} y}}{{dx^{3} }}\quad EI\left( {\cos ka,\sin ka} \right)\left[ \begin{gathered} - 4k^{3} C_{1} \quad - 4k^{3} C_{1} \hfill \\ \quad 0\quad \quad \quad \quad \quad 0 \hfill \\ \end{gathered} \right]\left( \begin{gathered} e^{ka} \hfill \\ e^{ - ka} \hfill \\ \end{gathered} \right) = - q_{0} a + D_{1}$$

43 $$EI\frac{{d^{2} y}}{{dx^{2} }}\quad EI\left( {\cos ka,\sin ka} \right)\left[ \begin{gathered} - 2k^{2} C_{1} \quad - 2k^{2} C_{1} \hfill \\ - 2k^{2} C_{1} \quad - 2k^{2} C_{1} \hfill \\ \end{gathered} \right]\left( \begin{gathered} e^{ka} \hfill \\ e^{ - ka} \hfill \\ \end{gathered} \right) = - \frac{{q_{0} }}{2}a^{2} + D_{1} a + D_{2}$$

44 $$EI\frac{dy}{{dx}}\quad EI\left( {\cos ka,\sin ka} \right)\left[ \begin{gathered} \quad 0\quad \quad \quad 0 \hfill \\ - 2kC_{1} \quad - 2kC_{1} \hfill \\ \end{gathered} \right]\left( \begin{gathered} e^{ka} \hfill \\ e^{ - ka} \hfill \\ \end{gathered} \right) = - \frac{{q_{0} }}{6}a^{3} + \frac{{D_{1} }}{2}a^{2} + D_{2} a + D_{3}$$

45 $$EIy(x)\quad EI\left( {\cos ka,\sin ka} \right)\left[ \begin{gathered} C_{1} \quad \; - C_{1} \hfill \\ - C_{1} \quad - C_{1} \hfill \\ \end{gathered} \right]\left( \begin{gathered} e^{ka} \hfill \\ e^{ - ka} \hfill \\ \end{gathered} \right) = - \frac{{q_{0} }}{24}a^{4} + \frac{{D_{1} }}{6}a^{3} + \frac{{D_{2} }}{2}a^{2} + D_{3} a + D_{4}$$

After determining $D_{1}$, $D_{2}$, $D_{3}$, and $D_{4}$, the deflection curve equation for segment $a \le x \le b$ is obtained as follows:

46 $$y(x) = \frac{1}{EI}\left( { - \frac{{q_{0} }}{24}x^{4} + \frac{{D_{1} }}{6}x^{3} + \frac{{D_{2} }}{2}x^{2} + D_{3} x + D_{4} } \right)$$

The following is obtained:

47 $$D_{1} = \frac{{\cos kx\left( {e^{ka} + e^{ - ka} } \right)q_{0} \left( {b - a} \right)}}{{\left[ {2 - \cos ka\left( {e^{ka} + e^{ - ka} } \right)} \right]}} + q_{0} a$$

48 $$D_{2} = \frac{{q_{0} \left( {b - a} \right)\left[ {\cos ka\left( {e^{ - ka} - e^{ka} } \right) - \sin ka\left( {e^{ka} + e^{ - ka} } \right) - 2ka\cos ka\left( {e^{ka} + e^{ - ka} } \right)} \right]}}{{2k\left[ {2 - \cos ka\left( {e^{ka} + e^{ - ka} } \right)} \right]}} - \frac{1}{2}q_{0} a$$

49 $$\begin{gathered} D_{3} = \frac{{q_{0} \left( {b - a} \right)\sin ka\left( {e^{ - ka} - e^{ka} } \right)}}{{2k^{2} \left[ {2 - \cos ka\left( {e^{ka} + e^{ - ka} } \right)} \right]}} - \frac{{D_{1} }}{2}a^{2} - D_{2} a + \frac{{q_{0} }}{6}a^{3} \hfill \\ = \frac{{q_{0} \left( {b - a} \right)\left[ {\sin ka\left( {e^{ - ka} - e^{ka} } \right) - k^{2} a^{2} \cos ka\left( {e^{ka} + e^{ - ka} } \right) - ka\cos ka\left( {e^{ - ka} - e^{ka} } \right) + ka\sin ka\left( {e^{ka} + e^{ka} } \right)} \right]}}{{2k^{2} \left[ {2 - \cos ka\left( {e^{ka} + e^{ - ka} } \right)} \right]}} + \frac{1}{6}q_{0} a^{3} \hfill \\ \end{gathered}$$

50 $$D_{4} = \frac{{q_{0} \left( {b - a} \right)[\cos ka\left( {e^{ka} - e^{ - ka} } \right) - \sin ka(e^{ka} + e^{ - ka} )]}}{{4k^{3} \left[ {2 - \cos ka\left( {e^{ka} + e^{ - ka} } \right)} \right]}} - \frac{{D_{1} }}{6}a^{3} - \frac{{D_{2} }}{2}a^{2} - D_{3} a + \frac{{q_{0} }}{24}a^{4}$$

The deformation deflection curve equation of the key layer beam structure in the nonfractured area of the coal rock mass in front of the initial mining stage is as follows:

51 $$\begin{gathered} \left\{ \begin{gathered} y\left( x \right) = \frac{{q_{0} (b - a)\left[ {\cos kx\left( {e^{kx} - e^{ - kx} } \right) - \sin kx\left( {e^{kx} + e^{ - kx} } \right)} \right]\quad }}{{4EIk^{3} \left[ {2 - \cos ka\left( {e^{ka} + e^{ - ka} } \right)} \right]}}0 \le x \le a \hfill \\ y(x) = \frac{1}{EI}\left( { - \frac{{q_{0} }}{24}x^{4} + \frac{{D_{1} }}{6}x^{3} + \frac{{D_{2} }}{2}x^{2} + D_{3} x + D_{4} } \right)a \le x \le b \hfill \\ \end{gathered} \right. \hfill \\ \hfill \\ \end{gathered}$$

The bending moment *m* at the midpoint is calculated using Eq. (17):

52 $$m + \int\limits_{0}^{a} {xq_{1} (x)} dx - \int\limits_{a}^{b} {xq_{0} (x)} dx = 0$$

53 $$m + \int\limits_{0}^{a} {xq_{1} (x)} dx - \frac{1}{2}q_{0} (b^{2} - a^{2} ) = 0$$

54 $$m = \frac{1}{2}q_{0} (b^{2} - a^{2} ) - 2k^{2} EIC_{1} \left( {\cos ka,\sin ka} \right)\left[ \begin{gathered} (k - 2ak)\quad ( - k - 2ak) \hfill \\ \quad 1\quad \quad \quad \quad \quad 1 \hfill \\ \end{gathered} \right]$$

55 $$C_{1} = \frac{{q_{0} (b - a)}}{{4EIk^{3} \left[ {2 - \cos ka(e^{ka} + e^{ - ka} )} \right]}}$$

The bending moment at the midpoint of the beam is as follows:

56 $$m = \frac{1}{2}q_{0} (b^{2} - a^{2} ) - \frac{{q_{0} (b - a)(\cos ka,\sin ka)\left[ \begin{gathered} (k - 2ak)\quad ( - k - 2ak) \hfill \\ \quad 1\quad \quad \quad \quad \quad 1 \hfill \\ \end{gathered} \right]\left( \begin{gathered} e^{ka} \hfill \\ e^{ - ka} \hfill \\ \end{gathered} \right)\quad }}{{2k\left[ {2 - \cos ka\left( {e^{ka} + e^{ - ka} } \right)} \right]}}$$

### Mechanical model of the coal rock mass during the initial mining period

#### Deformation mechanics model of the key layer elastic foundation boundary rock beam

**Fig. 5:**
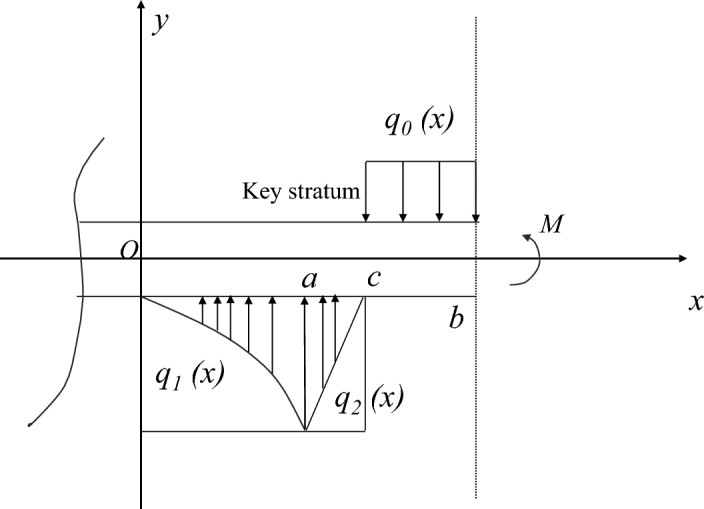
Deformation mechanics model of key strata on the elastic foundation boundary in the crushing zone of the rib.

The deformation mechanics model of the elastic foundation boundary rock beam in the key layer of the fractured zone in front of the initial mining face is presented in Fig. 5. This model is suitable for studying the deformation law of the roof after advancing a certain distance in the working face. The *x*-axis and *y*-axis of the model coordinate axis are set the same as those of the previous model. Point *a* is the horizontal distance of the compression area ahead of the working face affected by the mining structure of the key layer, excluding the crushing area, that is, the distance from the stress point of the original rock in front to the point where the coal wall of the working face begins to crush. Point *C* is at the coal wall of the working face. Point B is the distance from the midpoint of the goaf towards the original rock stress point in front of the working face. Then, 2(*b*-*c*) is the span of the roof rock beam.

Within the range of 0 ≤ *x* ≤ *a*, the key layer is subjected to a downwards load from the overlying rock layer and an upwards load from the elastic foundation in opposite directions, with a magnitude equal to the difference between the two, denoted as $q_{1} (x)$. Within the range of *a* ≤ *x* ≤ *c*, owing to the fragmentation of the elastic foundation, the elastic support force decreases, and the key layer is subjected to the transfer load of the overlying rock layer. Within the range of c ≤ x ≤ b, the key layer is subjected to a constant downwards uniformly distributed load $q_{0} (x)$ from the overlying rock layer.

#### Key layer beam deformation deflection curve for the elastic foundation boundary during the initial mining period

As the span of the working face increases, the roof begins to damage the coal wall in front of the working face, which is typically referred to as the relief zone. The load distribution function $q_{0} (x)$ during the coal fragmentation process in the depressurization zone is defined as follows:

57 $$q_{2} (x) = \frac{{q_{1} (a)}}{a - c}\left( {x - c} \right)$$

where $q_{1} (a)$ is the load at point *x* = *a.*

To distinguish it from $C_{1}$ in differential equations without fractured zones, it is represented by $C_{1}$ in differential equations with fractured zones present.

58 $$q_{1} (a) = \left( {coaka,\sin ka} \right)\left[ \begin{gathered} - 4k^{3} C_{1}^{\prime } \quad - 4k^{3} C_{1}^{\prime } \hfill \\ \quad 0\quad \quad \quad \quad 0 \hfill \\ \end{gathered} \right]\left( \begin{gathered} e^{ka} \hfill \\ e^{ka} \hfill \\ \end{gathered} \right)$$

According to the balance of forces:

59 $$EI\left[ {\frac{{d^{3} y}}{{dx^{3} }}} \right]_{0}^{a} + \int_{a}^{c} {q_{2} (x)} dx - \int_{c}^{b} {q_{0} (x)} dx = 0$$

According to Eq. (59):

60 $$EI\left[ {\frac{{d^{3} y}}{{dx^{3} }}} \right]_{0}^{a} + \frac{{q_{1} (a)}}{2}\left( {c - a} \right) - q_{0} (b - c) = 0$$

$C_{1}^{\prime }$ is determined from Eq. (60) as follows:

61 $$EI\left[ {\frac{{d^{3} y}}{{dx^{3} }}} \right]_{0}^{a} = \left( {coaka,\sin ka} \right)\left[ \begin{gathered} - 4EIk^{3} C_{1}^{\prime } \quad - 4k^{3} EIC_{1}^{\prime } \hfill \\ \quad 0\quad \quad \quad \quad 0 \hfill \\ \end{gathered} \right]\left( \begin{gathered} e^{ka} \hfill \\ e^{ - ka} \hfill \\ \end{gathered} \right) - \left( {1,0} \right)\left[ \begin{gathered} - 4EIk^{3} C_{1}^{\prime } \quad - 4k^{3} EIC_{1}^{\prime } \hfill \\ \quad 0\quad \quad \quad \quad 0 \hfill \\ \end{gathered} \right]\left( \begin{gathered} 1 \hfill \\ 1 \hfill \\ \end{gathered} \right) = \frac{{q_{1} (a)}}{2}\left( {c - a} \right) + q_{0} (b - c)$$

62 $$C_{1} ^{\prime} = \frac{{q_{1} (a)\left( {a - c} \right) + 2q_{0} (b - c)}}{{8EI\left[ {2 - \cos ka\left( {e^{ka} + e^{ - ka} } \right)} \right]}}$$

Under the condition of a fractured zone in front of the coal rock mass during the initial mining period, the key layer beam deformation deflection curve equation for the elastic foundation boundary is divided into three segments:

① The equation for the deflection curve in the range of 0 ≤ *x* ≤ *a* is as follows:

63 $$y(x) = \frac{{\left[ {q_{1} (a)\left( {a - c} \right) + 2q_{0} (b - c)} \right]\left[ {\cos kx\left( {e^{kx} - e^{ - kx} } \right) - \sin ka\left( {e^{kx} + e^{ - kx} } \right)} \right]}}{{8EIk^{3} \left[ {2 - \cos ka\left( {e^{ka} + e^{ - ka} } \right)} \right]}}$$

② The curve equation for the segment *a* ≤ *x* ≤ *c* is as follows:

64 $$EI\frac{{d^{4} y}}{{dx^{4} }} = \frac{{q_{1} (a)}}{a - c}\left( {x - c} \right)$$

65 $$EI\frac{{d^{3} y}}{{dx^{3} }} = \frac{{q_{1} (a)}}{2(a - c)}\left( {x - c} \right)^{2} + D_{1} ^{\prime}$$

66 $$EI\frac{{d^{2} y}}{{dx^{2} }} = \frac{{q_{1} (a)}}{6(a - c)}\left( {x - c} \right)^{3} + D_{1} ^{\prime}\left( {x - c} \right) + D_{2} ^{\prime}$$

67 $$EI\frac{dy}{{dx}} = \frac{{q_{1} (a)}}{24(a - c)}\left( {x - c} \right)^{4} + \frac{{D_{1} ^{\prime}}}{2}\left( {x - c} \right)^{2} + D_{2} ^{\prime}\left( {x - c} \right) + D_{3} ^{\prime}$$

68 $$EIy(x) = \frac{{q_{1} (a)}}{120(a - c)}\left( {x - c} \right)^{5} + \frac{{D_{1} ^{\prime}}}{6}\left( {x - c} \right)^{3} + \frac{{D_{2} ^{\prime}}}{2}\left( {x - c} \right)^{2} + D_{3} ^{\prime}\left( {x - c} \right) + D_{4} ^{\prime}$$

The curve equation of a beam in the a ≤ x ≤ c segment is as follows:

69 $$y(x) = \frac{1}{EI}\left[ {\frac{{q_{1} (a)}}{120(a - c)}\left( {x - c} \right)^{5} + \frac{{D_{1} ^{\prime}}}{6}\left( {x - c} \right)^{3} + \frac{{D_{2} ^{\prime}}}{2}\left( {x - c} \right)^{2} + D_{3} ^{\prime}\left( {x - c} \right) + D_{4} ^{\prime}} \right]$$

The coefficients of $D_{1} ^{\prime}$, $D_{2}$', $D_{3} ^{\prime}$, and $D_{4}$' in the definite formula are obtained below.

According to Eq. (65), it can be inferred that

70 $$\begin{gathered} D_{1} ^{\prime} = - EI4k^{3} C^{\prime}_{1} (\cos kx,\sin kx)\left[ \begin{gathered} 1\quad 1 \hfill \\ 0\quad 0 \hfill \\ \end{gathered} \right]\left( \begin{gathered} e^{kx} \hfill \\ e^{ - kx} \hfill \\ \end{gathered} \right) - \frac{{q_{1} (a)}}{2(a - c)}\left( {x - c} \right)^{2} \hfill \\ \quad \; = - EI4k^{3} C^{\prime}_{1} (\cos kx,\sin kx)\left( \begin{gathered} e^{kx} \hfill \\ e^{ - kx} \hfill \\ \end{gathered} \right) - \frac{{q_{1} (a)}}{2(a - c)}\left( {x - c} \right)^{2} \hfill \\ \quad \; = - EI4k^{3} C^{\prime}_{1} \cos kx(e^{kx} + e^{ - kx} ) - \frac{{q_{1} (a)}}{2(a - c)}\left( {x - c} \right)^{2} \hfill \\ \end{gathered}$$

According to Eq. (66), it can be inferred that

71 $$\begin{gathered} D_{2} ^{\prime} = 2k^{2} EIC_{1} ^{\prime}(\cos kx,\sin kx)\left[ \begin{gathered} - 1\quad 1 \hfill \\ - 1\quad - 1 \hfill \\ \end{gathered} \right]\left( \begin{gathered} e^{kx} \hfill \\ e^{ - kx} \hfill \\ \end{gathered} \right) - \frac{{q_{1} (a)}}{6(a - c)}\left( {x - c} \right)^{3} - D_{1} ^{\prime}\left( {x - c} \right) \hfill \\ \quad \; = 2k^{2} EIC_{1} ^{\prime}( - \cos kx - \sin kx,\cos kx - \sin kx)\left( \begin{gathered} e^{kx} \hfill \\ e^{ - kx} \hfill \\ \end{gathered} \right) - \frac{{q_{1} (a)}}{6(a - c)}\left( {x - c} \right)^{3} - D_{1} ^{\prime}\left( {x - c} \right) \hfill \\ \quad \; = 2k^{2} EIC_{1} ^{\prime}( - \cos kxe^{kx} - \sin kxe^{kx} + \cos kxe^{ - kx} - \sin kxe^{ - kx} ) - \frac{{q_{1} (a)}}{6(a - c)}\left( {x - c} \right)^{3} - D_{1} ^{\prime}\left( {x - c} \right) \hfill \\ \quad \; = 2k^{2} EIC_{1} ^{\prime}\left[ {\cos kx\left( {e^{ - kx} - e^{kx} } \right) - \sin kx\left( {e^{kx} + e^{ - kx} } \right)} \right] - \frac{{q_{1} (a)}}{6(a - c)}\left( {x - c} \right)^{3} - D_{1} ^{\prime}\left( {x - c} \right) \hfill \\ \end{gathered}$$

According to Eq. (67), it can be inferred that

72 $$\begin{gathered} D_{3} ^{\prime} = 2kEIC_{1} ^{\prime}(\cos kx,\sin kx)\left[ \begin{gathered} 0\quad \;0 \hfill \\ - 1\quad 1 \hfill \\ \end{gathered} \right]\left( \begin{gathered} e^{kx} \hfill \\ e^{ - kx} \hfill \\ \end{gathered} \right) - \frac{{D_{1} ^{\prime}}}{2}\left( {x - c} \right)^{2} - D_{2} ^{\prime}\left( {x - c} \right) - \frac{{q_{1} (a)}}{24(a - c)}\left( {x - c} \right)^{4} \hfill \\ \quad \; = 2kEIC_{1} ^{\prime}( - \sin kx,\sin kx)\left( \begin{gathered} e^{kx} \hfill \\ e^{ - kx} \hfill \\ \end{gathered} \right) - \frac{{D_{1} ^{\prime}}}{2}\left( {x - c} \right)^{2} - D_{2} ^{\prime}\left( {x - c} \right) - \frac{{q_{1} (a)}}{24(a - c)}\left( {x - c} \right)^{4} \hfill \\ \quad \; = 2kEIC_{1} ^{\prime}\sin kx\left( {e^{ - kx} - e^{kx} } \right) - \frac{{D_{1} ^{\prime}}}{2}\left( {x - c} \right)^{2} - D_{2} ^{\prime}\left( {x - c} \right) - \frac{{q_{1} (a)}}{24(a - c)}\left( {x - c} \right)^{4} \; \hfill \\ \end{gathered}$$

According to Eq. (68), it can be inferred that

73 $$\begin{gathered} D_{4} ^{\prime} = EIC_{1} ^{\prime}(\cos kx,\sin kx)\left[ \begin{gathered} 1\quad \; - 1 \hfill \\ - 1\quad - 1 \hfill \\ \end{gathered} \right]\left( \begin{gathered} e^{kx} \hfill \\ e^{ - kx} \hfill \\ \end{gathered} \right) - \frac{{q_{1} (a)}}{120(a - c)}\left( {x - c} \right)^{5} - \frac{{D_{1} ^{\prime}}}{6}\left( {x - c} \right)^{3} - \frac{{D_{2} ^{\prime}}}{2}\left( {x - c} \right)^{2} - D_{3} ^{\prime}\left( {x - c} \right) \hfill \\ \quad \; = EIC_{1} ^{\prime}\left[ {(\cos kx - \sin kx),( - \cos kx - \sin kx)} \right]\left( \begin{gathered} e^{kx} \hfill \\ e^{ - kx} \hfill \\ \end{gathered} \right) - \frac{{q_{1} (a)}}{120(a - c)}\left( {x - c} \right)^{5} - \frac{{D_{1} ^{\prime}}}{6}\left( {x - c} \right)^{3} - \frac{{D_{2} ^{\prime}}}{2}\left( {x - c} \right)^{2} - D_{3} ^{\prime}\left( {x - c} \right) \hfill \\ \quad \; = EIC_{1} ^{\prime}(\cos kxe^{kx} - \sin kxe^{kx} - \cos kxe^{ - kx} - \sin kxe^{ - kx} ) - \frac{{q_{1} (a)}}{120(a - c)}\left( {x - c} \right)^{5} - \frac{{D_{1} ^{\prime}}}{6}\left( {x - c} \right)^{3} - \frac{{D_{2} ^{\prime}}}{2}\left( {x - c} \right)^{2} - D_{3} ^{\prime}\left( {x - c} \right) \hfill \\ \quad \; = EIC_{1} ^{\prime}\left[ {\cos kx\left( {e^{kx} - e^{ - kx} } \right) - \sin kx\left( {e^{kx} + e^{ - kx} } \right)} \right] - \frac{{q_{1} (a)}}{120(a - c)}\left( {x - c} \right)^{5} - \frac{{D_{1} ^{\prime}}}{6}\left( {x - c} \right)^{3} - \frac{{D_{2} ^{\prime}}}{2}\left( {x - c} \right)^{2} - D_{3} ^{\prime}\left( {x - c} \right)\; \hfill \\ \end{gathered}$$

③ The curve equation of the beam in the *c* ≤ *x* ≤ *b* segment is as follows:

74 $$y(x) = \frac{1}{EI}\left( { - \frac{{q_{0} (a)}}{24}x^{4} + \frac{{D_{1}^{\prime \prime } }}{6}x^{3} + \frac{{D_{2}^{\prime \prime } }}{2}x^{2} + D_{3}^{\prime \prime } x + D_{4}^{\prime \prime } } \right)$$

The equation of the beam deflection curve under the condition of a fractured zone is as follows:

75 $$\left\{ \begin{gathered} y\left( x \right) = \frac{{\left[ {q_{1} \left( a \right)(a - c) + 2q_{0} \left( {b - c} \right)} \right]\left[ {\cos kx\left( {e^{kx} - e^{ - kx} } \right) - \sin kx\left( {e^{kx} + e^{ - kx} } \right)} \right]\quad }}{{8EIk^{3} \left[ {2 - \cos ka\left( {e^{ka} + e^{ - ka} } \right)} \right]}}0 \le x \le a \hfill \\ y(x) = \frac{1}{EI}\left[ {\frac{{q_{1} \left( a \right)}}{120(a - c)}(x - a)^{5} + \frac{{D_{1}^{\prime } }}{6}(x - c)^{3} + \frac{{D_{2}^{\prime } }}{2}(x - c)^{2} + D_{3}^{\prime } (x - c) + D_{4}^{\prime } } \right]a \le x \le c \hfill \\ y(x) = \frac{1}{EI}\left( { - \frac{{q_{0} }}{24}x^{4} + \frac{{D_{1}^{\prime \prime } }}{6}x^{3} + \frac{{D_{2}^{\prime \prime } }}{2}x^{2} + D_{3}^{\prime \prime } x + D_{4}^{\prime \prime } } \right)c \le x \le b \hfill \\ \end{gathered} \right.$$

#### Calculation of the bending moment *M* at the midpoint of the beam

76 $$M + \sum\nolimits_{j = 1}^{t} {\int\limits_{{x_{{s_{j} }} }}^{{x_{{e_{j} }} }} {xq(x)dx = 0} }$$

The above equation is reorganized as follows:

77 $$\int {xq_{1} (x)} dx = xQ_{1} - Q_{2}$$

78 $$M + \left[ {xQ_{1} - Q_{2} } \right]_{0}^{a} + \int\limits_{a}^{c} {xq_{2} (x)dx} - \int\limits_{c}^{b} {xq_{0} dx} = 0$$

79 $$M + \left[ {xQ_{1} - Q_{2} } \right]_{0}^{a} + \frac{{q_{1} (a)}}{6}\left( {c - a} \right)(c + 2a) - \frac{{q_{0} }}{2}(b^{2} - c^{2} ) = 0$$

① In the range of 0 ≤ *x* ≤ *a*, the bending moment $M(x)$ of the beam is determined by the following equation:

80 $$M(x) + \left[ {xQ_{1} - Q_{2} } \right]_{0}^{a} = 0$$

81 $$M(x) = - 2k^{2} EIC_{1} ^{\prime}(\cos kx,\sin kx)\left[ \begin{gathered} (k - 2xk)\quad \;( - k - 2xk) \hfill \\ \quad \quad 1\quad \quad \quad \quad 1 \hfill \\ \end{gathered} \right]\left( \begin{gathered} e^{kx} \hfill \\ e^{ - kx} \hfill \\ \end{gathered} \right)$$

② In the section *a* ≤ *x* ≤ *c*, the bending moment $M(x)$ of the beam is determined by the following equation:

82 $$M(x) + \left[ {xQ_{1} - Q_{2} } \right]_{0}^{a} + \frac{{q_{1} (a)}}{6}(x - a)(x + 2a) = 0$$

③ In the section c ≤ x ≤ b, the bending moment M(x) of the beam is determined by the following equation:

83 $$M(x) + \left[ {xQ_{1} - Q_{2} } \right]_{0}^{a} + \frac{{q_{1} (a)}}{6}(c - a)(c + 2a) - \frac{{q_{0} }}{2}(x^{2} - c^{2} ) = 0$$

where $$\left[ {xQ_{1} - Q_{2} } \right]_{0}^{a} = EIC_{1} ^{\prime}(\cos kx,\sin kx)\left[ \begin{gathered} 2k^{3} (1 - 2a)\quad \;2k^{3} ( - 1 - 2a) \hfill \\ \quad \quad 2k^{2} \quad \quad \quad \quad 2k^{2} \hfill \\ \end{gathered} \right]\left( \begin{gathered} e^{kx} \hfill \\ e^{ - kx} \hfill \\ \end{gathered} \right)$$.

The moment equation is as follows:

84 $$\left\{ \begin{gathered} M(x) = EIC_{1} ^{\prime}(\cos kx,\sin kx)\left[ \begin{gathered} 2k^{3} (2x - 1)\quad \;2k^{3} (1 + 2x) \hfill \\ \quad \quad - 2k^{2} \quad \quad \quad - 2k^{2} \hfill \\ \end{gathered} \right]\left( \begin{gathered} e^{kx} \hfill \\ e^{ - kx} \hfill \\ \end{gathered} \right) \hfill \\ M(x) = EIC_{1} ^{\prime}(\cos ka,\sin ka)\left[ \begin{gathered} 2k^{3} (2a - 1)\quad \;2k^{3} (1 + 2a) \hfill \\ \quad \quad - 2k^{2} \quad \quad \quad - 2k^{2} \hfill \\ \end{gathered} \right]\left( \begin{gathered} e^{ka} \hfill \\ e^{ - ka} \hfill \\ \end{gathered} \right) - \frac{{q_{1} (a)}}{6}\left( {x - a} \right)(x + 2a) \hfill \\ M(x) = EIC_{1} ^{\prime}(\cos ka,\sin ka)\left[ \begin{gathered} 2k^{3} (2a - 1)\quad \;2k^{3} (1 + 2a) \hfill \\ \quad \quad - 2k^{2} \quad \quad \quad - 2k^{2} \hfill \\ \end{gathered} \right]\left( \begin{gathered} e^{ka} \hfill \\ e^{ - ka} \hfill \\ \end{gathered} \right) - \frac{{q_{1} (a)}}{6}\left( {c - a} \right)(c + 2a) \hfill \\ \end{gathered} \right.$$

## Analysis of the deformation law of key layer beams at the boundary of the elastic foundation during the initial mining period

### Analysis of the deformation law of key layer beams in the nonfractured area of the coal rock mass in front of the initial mining stage working face

- (1) The analysis of the effect of key layer beam deformation on the coal rock mass in front of the working face under two boundary conditions in the 0 ≤ *x* ≤ *a* section is given here. Under other unchanged conditions, as the working face continues to advance, the span of the roof increases by 2(*b*-*a*) or 2(*b*-*c*), and the displacement of the centroid of the key layer inside the coal rock mass in the *y*-direction increases in front of the working face.
- (2) As the working face continues to advance, a fractured zone is formed in the coal body ahead. The displacement of the centroid of the key layer in the coal rock mass in the *y*-direction in front of the working face is greater than that in the absence of a fractured zone at the beginning of the advance.
- (3) The displacement of the centroid of the key layer in the coal rock mass along the y-direction is negatively correlated with the elastic coefficient k of the coal rock mass in front of the working face. The harder the coal body is, the smaller the displacement of the key layer. If the coal body is soft, the key layer displacement is large.
- (4) There is a negative correlation between the displacement of the centroid of the key layer in the coal rock mass along the y-direction and the bending stiffness *EI* in front of the working face.
- (5) The deformation law of the key strata in the goaf of the working face is as follows: the displacement of the centroid of the key strata along the y-direction is negatively correlated with the bending stiffness *EI*, and the displacement curve of the centroid of the key layer along the y-direction shows a polynomial variation pattern.

### Solving mechanical model examples

#### Selection of relevant parameters

On the basis of the geological data of the mine and onsite measured data, values are taken for each parameter in the model.

*K* is the elastic basic coefficient:

85 $$k = \frac{{E_{1} E_{2} }}{{E_{2} h_{1} + E_{1} h_{2} }}$$

where *h*_*1*_ is the direct top thickness, m; *h*_*2*_ is the coal seam thickness, m; *E*_*1*_ is the direct top elastic modulus, MPa; *E*_*2*_ is the coal seam elastic modulus, GPa; and *K* is the boundary elastic basic coefficient, MN/m^3^.

The direct top is sandy mudstone, with an h1 value of 6.6 m and an *E*_*1*_ value of 1.6 × 10^4^ MPa; the coal seam is 3 + 4 # coal, with an *h*_*2*_ value of 4.6 m and an *E*_*2*_ value of 2.7 × 10^4^ MPa.

E and I are the elastic modulus and moment of inertia of the critical layer, respectively. According to the geological data of the Shaqu Mine and related data on ore pressure and separation, the key layer of the overlying rock layer of 3 + 4 # coal is medium sandstone with a thickness of 4.5 m. The elastic modulus E is 4.25 × 104 MPa, and the beam width is set to 40 m. The moment of inertia I is calculated to be 303.75 m4.

#### Boundary condition values

The specific boundary conditions of the unbreakable zone are shown in Table 1.

**Table 1:** Deflection curve equation parameters under the boundary of the noncrushing zone of the rib.

Model parameter	Value	Unit		Model parameter	Value	Unit
k	0.17	MN/m^3^		*E*	4.25 × 10^4^	MPa
a	58	m		*I*	303.75	m^4^
b	73	m		q_0_	12.845	MPa

The specific boundary conditions of the fractured zone are shown in Table 2.

**Table 2:** Deflection curve equation parameters under the boundary of the crushing zone of the rib.

Model parameter	Value	Unit		Model parameter	Value	Unit
*k*	0.17	MN/m^3^		*E*	4.25 × 10^4^	MPa
*a*	58	m		*I*	303.75	m^4^
*b*	81	m		*q*_*0*_	12.845	MPa
*c*	66	m		*q*_*1*_*(a)*	38.535	MPa

#### Results analysis

**Fig. 6:**
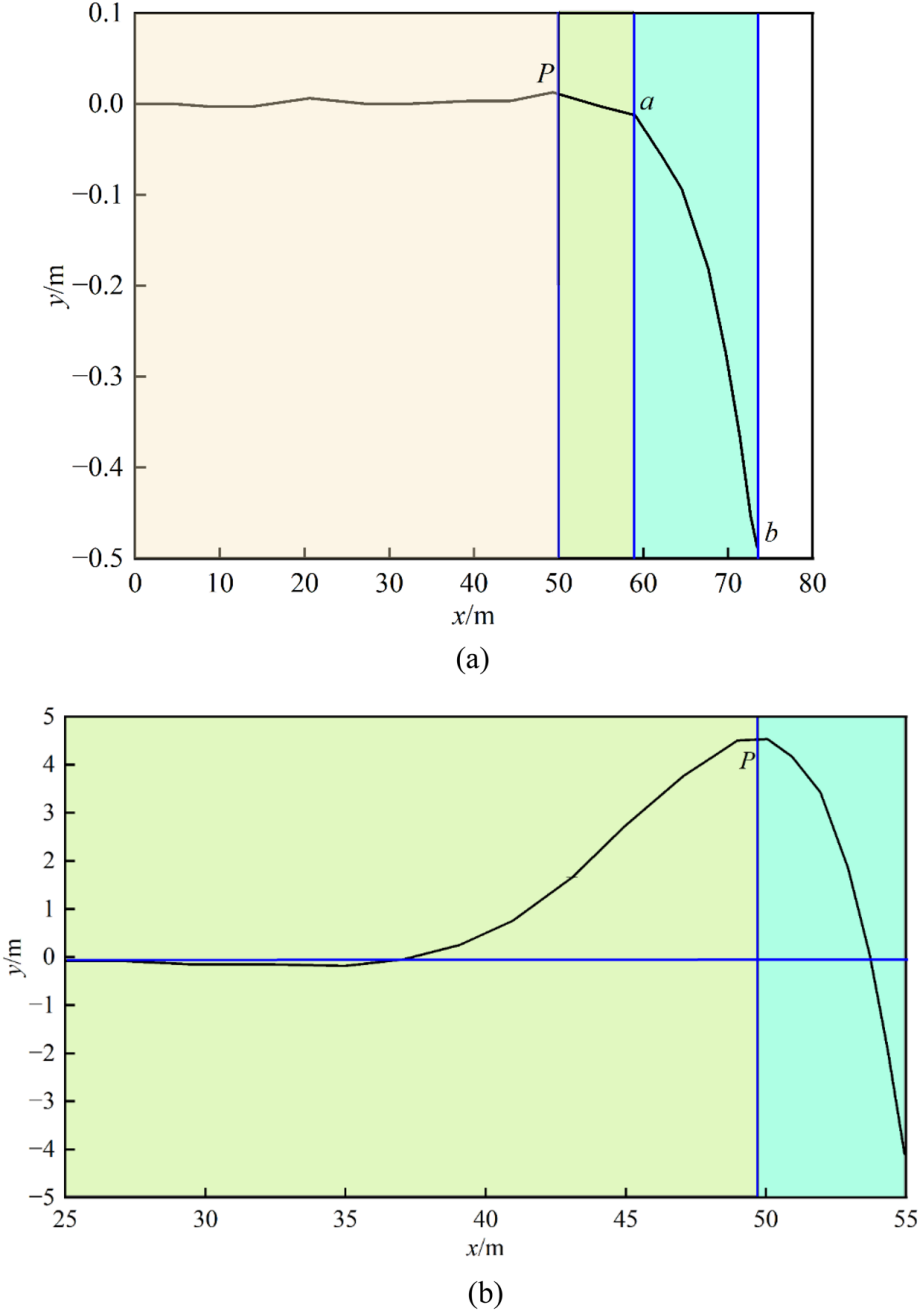
Deflection curve diagram of key strata deformation in the noncrushing zone of the rib. (**a**) Full deflection curve. (**b**) Deflection curve of the fluctuation Sect. (25 ~ 55 m).

According to Fig. 6, the vertical displacement of the key layer above the solid coal in front of the coal wall alternately increases and decreases towards the deep part of the coal rock mass, with the peak amplitude gradually decreasing and approaching zero. The maximum bending moment of the beam before fracture occurs at point b in the middle section of the goaf, and the peak deflection curve of the key layer appears at point *p* in the coal body near the working face. The bending moment value at this point is second only to that at point *b*, which corresponds to the stress concentration point of the advanced support pressure of the working face. The phenomenon of “upwards lifting” of the critical layer in this area is caused by the obstruction of load transfer during the bending deformation process of the beam. When the deformation of the beam reaches the breaking limit, the critical layer breaks at point *b*. However, the fractured rock blocks form a hinged structure through line or point contact. As the span of the working face continues to increase, the rock blocks rotate, become unstable, and eventually collapse^30,31^. After the critical layer breaks at point *b*, it becomes a cantilever beam, which is equivalent to removing the bending moment *M* in this mechanical model. Moreover, under the joint action of load transfer, the cantilever beam part of the critical layer produces a “rebound” effect, and the stress concentration point transfers to the deep part of the coal rock mass. The influence distance of advanced mining on the working face is positively correlated with the bending stiffness *EI* of the key layer. During the bending and sinking process of the key layer, loads are transmitted to the front coal rock mass, and disturbance is exerted on the evolution of mining-induced fractures in the coal rock mass.

**Fig. 7:**
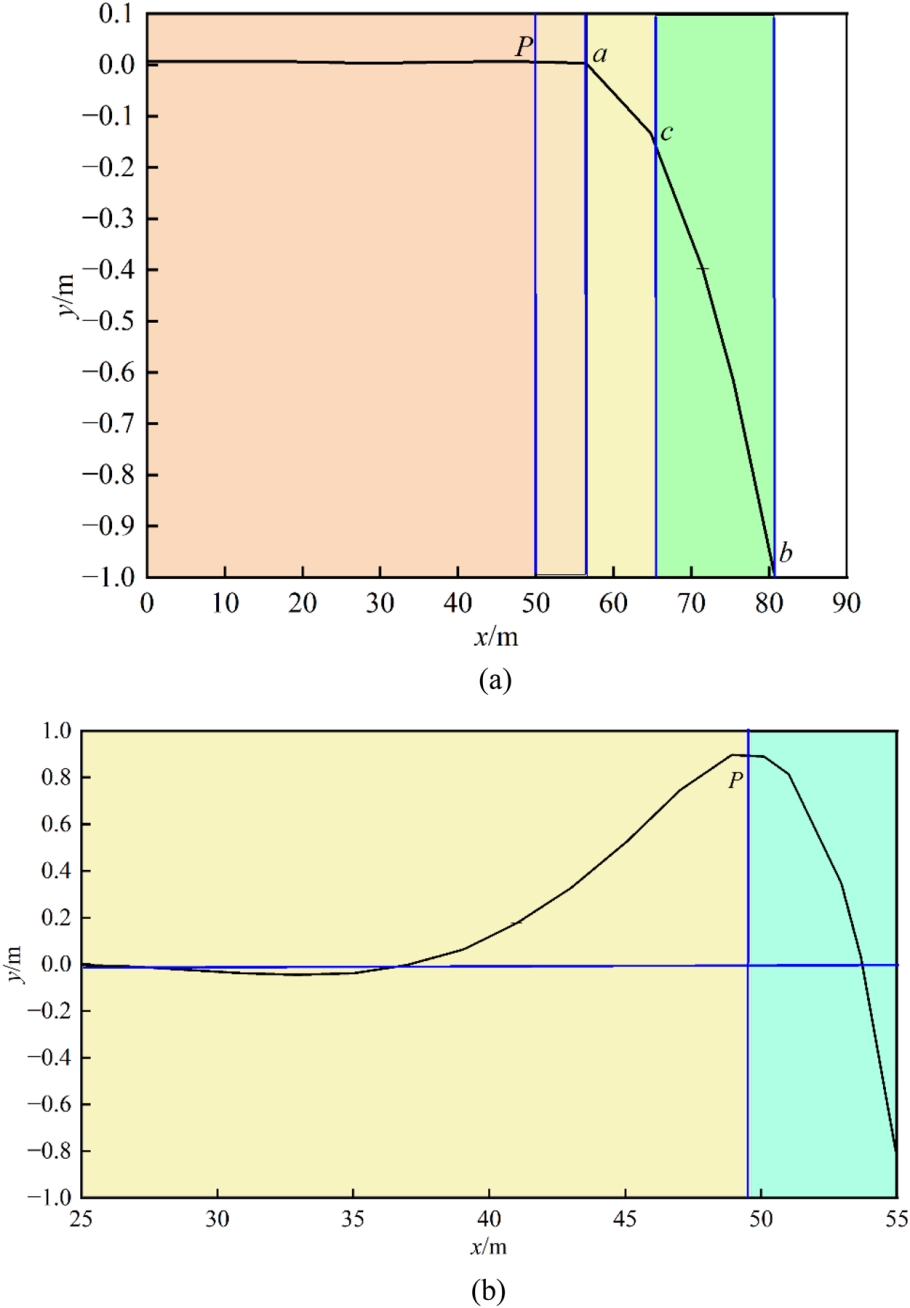
Deflection curve diagram of key strata deformation in the crushing zone of the rib. (**a**) Full deflection curve. (**b**) Deflection curve of the fluctuation Sect. (25 ~ 55 m).

As the working face advances, the span of the key layer beam gradually increases, and when it reaches the deformation limit, it breaks at the middle position, which represents the maximum bending moment according to Fig. 7. However, owing to the front and rear support points of the beam in the coal rock mass, which are the peak bending moment points at the “upwards uplift” mentioned earlier, smooth bending deformation is maintained at this time. At the moment of critical layer fracture, the fractured rock blocks exist in a hinged beam structure that maintains relative stability. The overhanging part that has just become a cantilever beam experiences a brief “rebound” and then begin to bend and deform under the action of the overlying rock layer. At this time, the maximum bending moment of the key layer cantilever beam is at a certain position inside the coal rock mass of the working face.

This model is applicable for simulating the elastic supporting characteristics of the rock layers beneath the coal seam. In the mining of high-gas and low-permeability coal seams, the bottom coal and rock layers possess certain elastic properties, which have a significant impact on the stability of the key layer^32,33^. By using field experiments and measurement data to determine the elastic modulus and Winkler foundation coefficient, the model can accurately reflect the actual working conditions. This assumption is particularly applicable in the initial mining stage, which helps to predict the settlement and deformation behavior of the key layer, thereby guiding safe mining and disaster prevention. During the initial mining stage, the deformation of the key layer mainly occurs in the middle part of the coal and rock layer in front of the working face. By establishing a bending deformation model of the beam, the deflection curve and bending moment distribution of the key layer can be obtained. This assumption provides a theoretical basis for quantitative analysis of the deformation of the key layer, and helps to evaluate the stability and safety under different mining conditions. For example, in two cases with boundary conditions of fractured coal wall and unfractured coal wall, the model can simulate the deformation behavior of the key layer respectively, providing important references for actual engineering.

## Conclusion

- (1) A key layer mechanics model was established, and the deflection curve equation, bending moment equation, and quantitative relationship between the working face advancement and the influence distance of mining advance were given for the deformation of the key layer.
- (2) The distance affected by advanced mining during the initial mining period is positively correlated with the bending stiffness *EI* of the key layer. During the bending deformation process of the critical layer, the load is transmitted to the coal rock mass in front of the working face, and it has a disturbance effect on the evolution of mining-induced fractures in the coal rock mass.
- (3) The deformation law of the key strata in the goaf of the working face is as follows: the displacement of the centroid of the key strata along the *y*-direction is negatively correlated with the bending stiffness *EI*, and the displacement curve of the centroid of the key layer along the *y*-direction shows a polynomial variation pattern.

## Data Availability

The data that support the findings of this study are available from the corresponding author H.Y.L.
